# Intrinsic network activity reflects the ongoing experience of chronic pain

**DOI:** 10.1038/s41598-021-01340-0

**Published:** 2021-11-08

**Authors:** Pauline Jahn, Bettina Deak, Astrid Mayr, Anne Stankewitz, Daniel Keeser, Ludovica Griffanti, Viktor Witkovsky, Stephanie Irving, Enrico Schulz

**Affiliations:** 1 Department of Neurology, University Hospital LMU, Ludwig-Maximilians-Universität München, Munich, Germany; 2 Department of Medical Psychology, Ludwig-Maximilians-Universität München, Munich, Germany; 3 Department of Psychiatry, University Hospital LMU, Ludwig-Maximilians-Universität München, Munich, Germany; 4 Department of Radiology, University Hospital LMU, Ludwig-Maximilians-Universität München, Munich, Germany; 5 Wellcome Centre for Integrative Neuroimaging, Department of Psychiatry and Nuffield Department of Clinical Neurosciences, University of Oxford, Oxford, UK; 6 Department of Theoretical Methods, Institute of Measurement Science, Slovak Academy of Sciences, Bratislava, Slovak Republic

**Keywords:** Sensory processing, Chronic pain

## Abstract

Analyses of intrinsic network activity have been instrumental in revealing cortical processes that are altered in chronic pain patients. In a novel approach, we aimed to elucidate how intrinsic functional networks evolve in regard to the fluctuating intensity of the experience of chronic pain. In a longitudinal study with 156 fMRI sessions, 20 chronic back pain patients and 20 chronic migraine patients were asked to continuously rate the intensity of their endogenous pain. We investigated the relationship between the fluctuation of intrinsic network activity with the time course of subjective pain ratings. For chronic back pain, we found increased cortical network activity for the salience network and a local pontine network, as well as decreased network activity in the anterior and posterior default mode network for higher pain intensities. Higher pain intensities in chronic migraine were accompanied with lower activity in a prefrontal cortical network. By taking the perspective of the individual, we focused on the variability of the subjective perception of pain, which include phases of relatively low pain and phases of relatively high pain. The present design of the assessment of ongoing endogenous pain can be a powerful and promising tool to assess the signature of a patient’s endogenous pain encoding.

## Introduction

In recent years neuroimaging studies on pain have largely shifted their focus to the analysis of intrinsic functional networks. These studies have analysed the spatial characteristics of patients’ cortical maps in comparison to healthy control subjects and detected a number of networks that are altered in chronic pain patients^1,2^.

Key findings have been reported for the default mode network (DMN). For example, when comparing patients with chronic back pain (CBP) and patients with chronic regional pain syndrome (CRPS) to healthy controls, Baliki et al. found significant local connectivity differences in brain regions within the DMN. CBP patients showed decreased connectivity to the medial prefrontal cortex and increased connectivity to the precuneus and the right lateral parietal region, as well as to regions outside the DMN, such as decreased connectivity to the anterior cingulate cortex and the left anterior insula/inferior-frontal gyrus^2^. For the whole brain DMN weighted map, Loggia et al. found a stronger effect in patients with CBP compared to healthy subjects in the medial prefrontal cortex as well as in the left inferior parietal lobule. Furthermore, the authors revealed a stronger DMN-insula connectivity for the patient group^3^. A study on chronic migraine found the opposite effect^1^.

Similarly, the salience network (SN) of the brain has received considerable interest due to its higher activity when attending to pain^4^. Kim et al. found a significant cluster of increased functional connectivity in the SN of patients with CRPS compared with healthy controls^5^. Likewise, Van Ettinger-Veenstra showed increased connectivity in the SN in patients with chronic widespread pain when compared to controls, particularly in the left anterior insula/superior temporal gyrus^6^. Similar to the DMN, chronic migraineurs also showed a contrary result by exhibiting a decreased effect for the SN^1^.

Here, we aimed to overcome previous approaches to analyse stationary maps of intrinsic networks, which cannot fully take into account ongoing dynamics of pain and network activity. As a major advantage, we addressed non-stationary network activity and assessed how the activity of intrinsic networks is evolving over time. Furthermore, we explored whether this evolving time course of intrinsic networks reflects the fluctuating intensity of the subjective experience of chronic pain. Consequently, by using Linear Mixed Effect Models (LME), we related the variable strength of intrinsic network activity to the varying subjective experience of endogenous pain in two frequent groups of patients: chronic back pain (CBP) and chronic migraine (CM). Due to the exploratory nature of this study, the unprecedented statistical approach, and the fact that the study is not pre-registered, we would not specify any dedicated hypothesis.

## Results

### Questionnaires

Patients were characterised using the German Pain Questionnaire (Deutscher Schmerzfragebogen)^7^ and the German-version of the Pain Catastrophising Scale (PCS)^8^. The pain intensity describes the average pain in the last four weeks from zero to 10 with zero representing no pain and 10 indicating maximum imaginable pain (please note that this scale differs from the one used in the fMRI experiment). The Depression, Anxiety and Stress Scale (DASS) was used to rate depressive, anxiety, and stress symptoms with a cutoff of 10 for depression and stress and 6 for anxiety^9^. A total PCS score of 30 represents a clinically-relevant level of pain catastrophising^8^.

The mean pain intensity specified in the questionnaires was 5 ± 2 for CBP and 5 ± 1 for CM (scale ranging from 0 to 10). The mean duration of the chronic pain was 10 ± 7 years for CBP and 15 ± 12 years for CM. The scores for the PCS were 17 ± 10 for CBP and 21 ± 10 for CM. For CBP the depression scale was 4 ± 3, the anxiety scale 3 ± 2 and the stress scale 7 ± 4. For CM the depression scale was 3 ± 3, the anxiety scale 3 ± 4 and the stress scale 6 ± 4 (all results given as mean ± standard deviation). Please see Supplementary Tables 1 and 2 for detailed patient characteristics and questionnaire data. A study on healthy subjects found similar results. A large sample of 1794 participants reported scores for depression of 3 ± 4, for anxiety of 2 ± 3 and for stress of 5 ± 4^10^. None of the patients reported any psychiatric comorbidity.

### Behavioural data

Patients were asked to continuously rate the pain during the recording of cortical activity using echo-planar imaging (EPI). The average pain ratings were variable between recording sessions. For CBP and CM we found an average rating of 39 (± 14) and 40 (± 15), respectively. The pain ratings within each session were substantially fluctuating, as reflected by a high variance over the 25 min of recording: σ^2^ = 109.3 (± 126.6) for CBP and σ^2^ = 93.3 (± 62.8) for CM. In general, we found a minimal positive slope of 0.13 (± 0.41; change of rating unit per minute) for CBP and of -0.05 (± 0.37) for CM (all mean ± standard deviation). The high similarity of pain ratings along the time course of the recording is also shown by the largely overlapping within-session distribution of pain ratings for the first and second half of the recording, which amounts to 74 ± 17% for CBP and to 71 ± 13% for CM (number of identical ratings between the first and second half divided by the number of all ratings of one half). A potential constantly rising pain, which we would have excluded from the present study, would have resulted in 0% overlap.

### Imaging results: chronic low back pain

**Figure 1:**
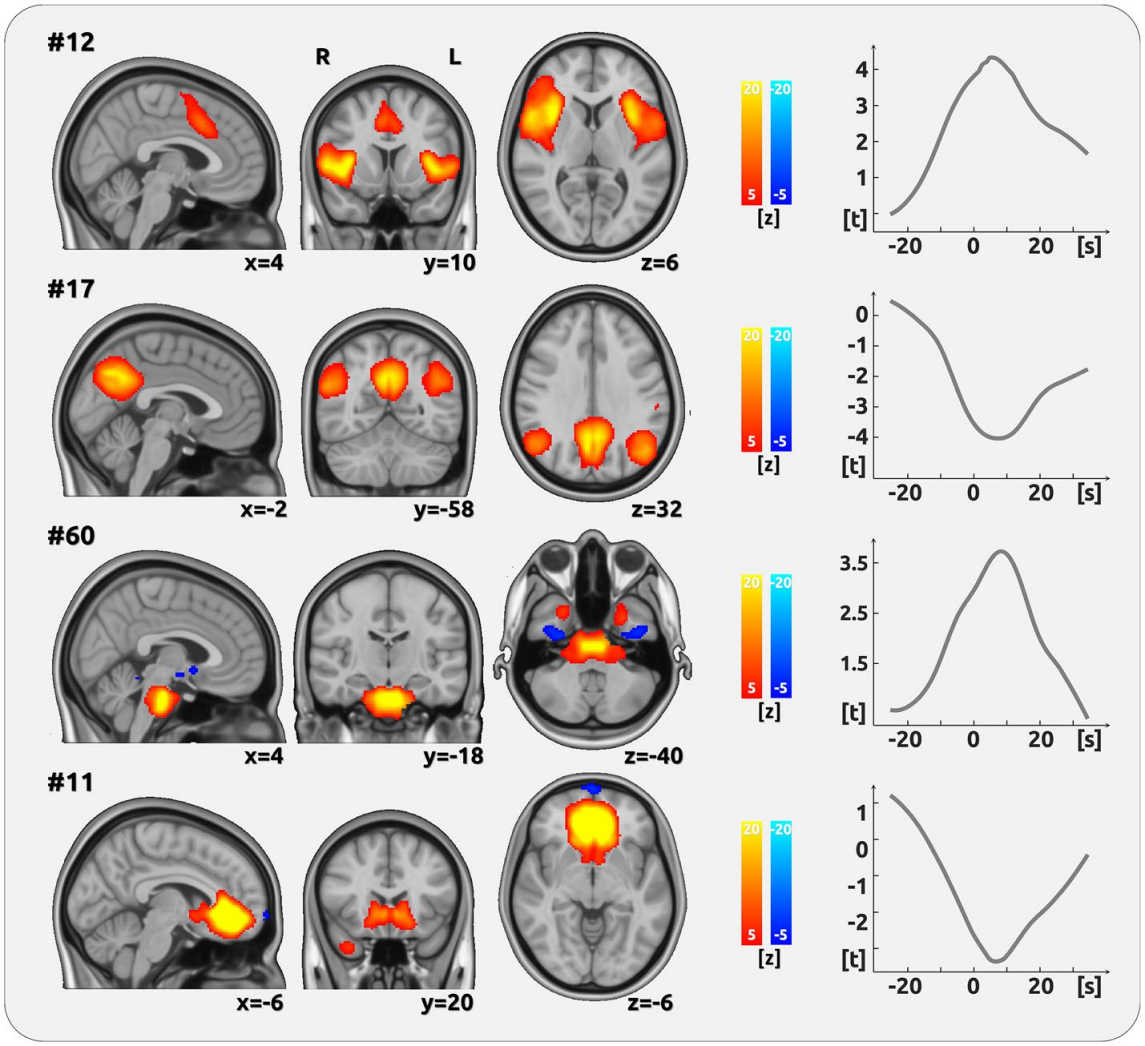
Encoding of pain amplitude for CBP. The figure shows the four networks whose time course exhibited a significant positive relationship with the time course of pain intensity (AMP). The cortical maps on the left side show the spatial characteristics of the networks and are the result of the Melodic ICA. Z-scores were also determined by Melodic and depict the contribution of each voxel to the respective network. The right side exhibits the analysis of the component time courses. The time courses, which are the output from the dual regression, were related to the time course of pain ratings using LMEs. The graph shows the results of the shifts between − 25 and 35 s in relation to the current rating (0 s). One significant network component (#12) essentially comprises the insular cortex and the posterior part of the anterior cingulate cortex. A further component with a positive relationship with pain is mainly related to pontine activity (#60). Negative relationships were found for the posterior (#17) and the anterior part of the DMN (#11).

We analysed the pain-related activity of intrinsic functional networks for CBP (Fig. 1, Table 1). We found two cortical networks whose time course exhibited a significant positive relationship with the time course of pain intensity (pain amplitude - AMP). We found a positive relation for pain intensity with the SN, which includes the insular cortex, the frontal operculum, the ACC, the paracingulate cortex (component 12), as well as with a local pontine network (component #60). Further networks, which included the anterior (component #11, aDMN) and the posterior default mode network (component #17, pDMN), exhibited negative relationships. The pDMN includes the angular cortex, the lateral occipital cortex, the precuneus, and the PCC. The pDMN comprises the nucleus accumbens, the nucleus caudatus, the subcallosal cortex, the orbitofrontal cortex, the frontal pole, and the temporal pole. We did not find any effect for the change of the direction of pain intensity (slope - SLP). The spatial correlation of the components with the rsn70 dataset of Smith and colleagues^11^ exhibited a large overlap (pDMN: *r* = 0.82 with #31; aDMN: *r* = 0.53 with #7; SN: *r* = 0.67 with #20).

**Table 1:** Intrinsic brain networks that encode the intensity of CBP.

#	Main regions of the intrinsic brain network	t-value	*p*-value (PALM)
12	Insular Cortex, Frontal Operculum, Anterior Cingulate Cortex, Paracingulate Cortex (salience network)	4.6	0.001
17	Angular Cortex, Lateral Occipital Cortex, Precuneus, Posterior Cingulate Cortex (posterior default mode network)	− 4	0.014
60	Brainstem (Pons)	3.8	0.039
11	Ncl. accumbens, Ncl. caudatus, Subcallosal Cortex, Orbitofrontal Cortex, Frontal Pole, Temporal Pole (anterior default mode network)	− 3.7	0.040

The t-values represent the relationship between the fluctuating subjective experience of CBP and the time course of component activity (see Fig. 1, right).

### Imaging results: chronic migraine

**Figure 2:**
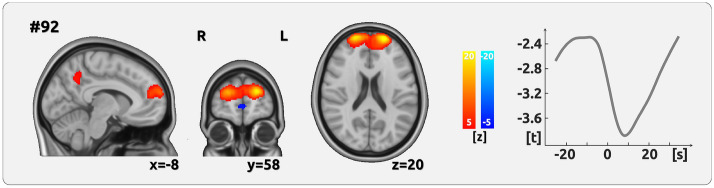
Encoding of pain amplitude for CM. The figure shows the network whose time course exhibited a significant negative relationship with the time course of pain intensity (AMP). The cortical maps on the left side show the spatial characteristics of the networks and are the result of the Melodic ICA. Z-scores were also determined by Melodic and depict the contribution of each voxel to the respective network. The right side exhibits the analysis of the component time courses. The time courses, which are the output from the dual regression, were then related to the time course of pain ratings using LMEs. The graph shows the results of the shifts between − 25 and 35 s in relation to the current rating (0 s). The network essentially comprises the prefrontal cortex.

The results for the pain-related activity of intrinsic functional networks for CM reveal a different picture. The time course of activity in a network that includes the frontal pole, the precuneus and the PCC (prefrontal- precuneus network, component #92, Fig. 2, Table 2) exhibited a significant negative relationship with the time course of pain intensity (AMP). We did not find any effect for the change of the direction of pain intensity (slope - SLP).

**Table 2:** Intrinsic brain network that encodes the intensity of CM.

#	Main regions of the intrinsic brain network	t-value	*p*-value (PALM)
92	Frontal Pole, Precuneus, Posterior Cingulate Cortex (prefrontal-precuneus network)	− 3.9	0.017

The t-value represents the relationship between the fluctuating subjective experience of CM and the time course of component activity (see Fig. 2, right).

## Discussion

We revealed variable, non-stationary network activity to represent phases of relatively high and phases of relatively low pain. For higher pain intensities, chronic back pain exhibited an increased network activity for the SN and a local pontine network, as well as decreased network activity in the anterior and posterior DMN. Higher pain intensities in chronic migraine are accompanied by lower activity in a bilateral prefrontal network. CBP patients used other networks than CM patients to encode the intensity of pain. Due to the fact that we have investigated only two pain diseases, we would not assume a specificity of these networks for any pain disease. Furthermore, the networks found in the present study are not specific for the encoding of pain intensity either; they are found to be relevant for a large number of cortical functions and conditions. The dynamic aspects of pain can be considered as the core of the patient's suffering from pain disease. Cortical networks that reflect these dynamic processes may indicate treatment success: patients with higher levels of endogenous pain are relieved by therapies that reduce pain intensity to tolerable levels^12,13^.

### Chronic low back pain

#### Default mode network

The DMN is thought to represent task-negative experimental periods in the absence of task demands^14^, allowing subjects to fall into a relaxed introspective or self-referencing mode^15,16^. We found a negative relationship between the DMN (anterior and posterior parts) and pain intensity in CBP patients, which may represent the gradual levels a subject can transfer themselves into a certain *relaxed-introspective* mental condition^17^. Phases of low pain are likely to allow this mode rather than phases of high pain, which may require more attention to the experienced chronic pain input. The DMN has been suggested to be controlled by a network consisting of the anterior insular cortex and the ACC which is largely overlapping with the SN in the present study (see below)^18,19^. The DMN has been previously shown to be affected in chronic back pain and other chronic pain states^2,20^. These DMN alterations were interpreted to represent dysfunctions related to a maladaptive physiology^2,21^. However, the present findings indicate a different interpretation; they are also in line with previous research on the brain-state dependent role of the DMN^22^, reflected in the present study by phases of high (~ high-amplitude DMN) and low pain (~ low-amplitude DMN).

#### Salience network

For chronic low back pain patients, we found a pain-related network that consists of the entire insular cortex and the posterior part of the cingulate cortex. The regions of these networks have - on the one hand - been found in numerous studies on the processing of pain^23,24^ and - on the other hand - been interpreted as SN in resting-state studies^25,26^. In fact, both aspects are not necessarily mutually exclusive^27^. The combined activity of both regions could represent the unspecific (salience) aspect that has been suggested to contribute to the processing of several sensory modalities, including pain^28^. Therefore, the present data suggest a similar mechanism for the processing of chronic low back pain intensity, whose primary and common aspect appears to be its saliency.

#### Pontine network

We found a positive relationship between the local pontine network activity and the intensity of CBP. There is less evidence of a pontine contribution to the processing of chronic low back pain so far. This might be caused by the various methodological differences between the present study and previous work and a general neglect of BOLD fluctuations in the white matter^29^. For the present study, the time course of ventral pontine BOLD activity shows a clear and specific effect at the moment of the pain rating as well as a dropping relationship before and afterwards. The contribution of the (dorsal) pons has been shown in a study that investigated structural changes in chronic back pain patients compared to healthy controls^30^.

### Chronic migraine

#### Prefrontal-precuneus network

For chronic migraine patients, we found a prefrontal-precuneus network to negatively relate to the ongoing fluctuations of pain intensity. For frontal regions, previous studies found structural changes^31–33^, as well as decreased functional connectivity^1^ in patients with chronic migraine compared to healthy controls.

The absence of any effect in pain-related areas may reflect the complexity of migraine^34^. While the detailed pathophysiological mechanisms for the chronification of migraine are not fully understood^35,36^, the current data with multiple recordings can open a window to explore stable and individually specific signatures for each patient.

#### Hypothalamus

We found no significant involvement of the hypothalamus in any of the functional networks. This absence of hypothalamic effect is suggested to be related to two aspects. *First*, we did not find any network that comprises a substantial and distinct portion of the hypothalamus. As the algorithm of the ICA requires large fluctuations of cortical signals, this might not be the case for hypothalamic signals, which exhibit rather small and noise-sensitive amplitude fluctuations. *Secon*d, the hypothalamus was found to be critically involved in episodic migraineurs during the headache^37^ as well as during the premonitory phase^38^, or in chronic migraine patients in response to experimentally applied pain^39^. In contrast, we were exploring the fluctuating endogenous head pain intensity in chronic migraineurs outside an attack. Therefore, due to its involvement in cyclic aspects of migraine, the hypothalamus is believed to serve as an attack trigger rather than to encode the intensity of head pain^40^. Therefore, the effects of previous studies are not directly comparable with the present finding due to methodological differences, i.e. subjective experience of endogenous pain vs. experimentally applied pain events, pain encoding vs. pain contrasted to baseline, and intrinsic network activity vs. event-related design.

### Fluctuations of network activity in chronic pain

Previous studies investigating intrinsic networks found chronic pain patients had impairments in the DMN^2,3,41^ and the SN^4–6^ when compared with healthy controls. Specifically for chronic migraine, decreased intrinsic network connectivity has also been found for the frontal executive control network^1^. Common to all of these studies is the voxel-wise analysis on individual maps of intrinsic components. These maps are interpreted as representing the *stationary* strengths of functional connectivity across the entire recording period^42^. While voxel-wise analyses allow to evaluate local changes in functional connectivity within a network, significant effects in voxels outside of the main regions of the network are often difficult to interpret. Moreover, a mere focus on cortical maps leaves open the question whether there are different physiological or cognitive states during peaks and troughs of the network time series. Therefore, the stationary network analyses may not represent the core pain experience of the patients, which is characterised by its fluctuating amplitude of pain intensity, unpleasantness, and distress.

The present study is focused on a rather neglected aspect of network activity, which is the *non-stationary* and fluctuating time course of intrinsic network activity. This time course is dominated by the voxels in the centre of the component maps due to their higher statistical weights. Here, we are able to analyse and interpret the peaks and troughs of the ongoing intrinsic network activity by relating it to the ongoing and variable subjective experience of pain. Although we are analysing intrinsic networks, this study is no resting-state study.

### Summary and outlook

The present investigation has targeted how the ongoing perception of chronic pain is subserved in the human brain. By taking into account the perspective of the individual, we focussed on the processes that matter for each patient, which are phases of relatively low pain and more straining phases of relatively high pain. We pursued a group-wise statistical approach in order to reliably assess the encoding of chronic pain in CM and CBP.

The present investigation also permits a glance into potential future investigations. The different intrinsic networks of both diseases and their relationship to ongoing pain may trigger new research on the involvement of cortical processes for the development of certain chronic pain syndromes. Due to the novelty of the present investigation, future studies are needed to explore which cognitive (e.g. attention) and physiological variations (e.g. autonomic responses) affect the encoding of chronic pain intensity in intrinsic networks. In addition, follow-up studies on other pain syndromes are needed in order to explore whether the current findings can be generalised.

Although we could not take any individual variations into consideration, there are current advances to examine individual trajectories of chronic pain experiences. These trajectories can be unique to the individual and the present design of ongoing assessment of the endogenous pain could be a powerful and promising tool for these analyses. With an accompanying and repeated assessment of a patient’s endogenous pain encoding over weeks or months, future therapies would be enabled to track a patient’s current cortical pattern of pain encoding; changes of these patterns may indicate the success of therapeutic interventions. A tailored medical treatment that is based on the changes of a patient’s endogenous pain signature could be a promising outlook into the future of medicine.

## Materials and methods

### Participants

The study included 20 patients diagnosed with chronic back pain (CBP—16 female; aged 44 ± 13 years) and 20 patients with chronic migraine without aura (CM - 18 female; aged 34 ± 13 years). All participants gave written informed consent. The study was approved by the Ethics Committee of the Medical Department of the Ludwig-Maximilians-Universität München and conducted in conformity with the Declaration of Helsinki.

CBP patients were diagnosed according to the IASP criteria (The International Association for the Study of Pain)^43^, which includes a disease duration of more than 6 months (mean CBP: 10 ± 7 years). All patients were seen in a specialised pain unit. CM patients were diagnosed according to the ICHD-3^44^, defined as a headache occurring on 15 or more days/month for more than 3 months, which, on at least 8 days/month, has the features of migraine headache (mean CM: 15 ± 12 years). All CM patients were seen in a tertiary headache centre.

All patients were permitted to continue their pharmacological treatment at a stable dose (Supplementary Tables 1 and 2). The patients did not report any other neurological or psychiatric disorders, or had contraindications for an MRI examination. Patients who had any additional pain were excluded. CBP patients with no pain on the day of the measurement were asked to return on a different day. CM patients were tested on a day with headache but not on a day with a migraine attack. Patients were compensated with 60€ for each session.

A total of nine patients were excluded: two patients developed additional pain during the study, the pain ratings of five patients were constantly increasing or decreasing throughout the pain rating experiment, and two patients were unable to comply with study requests (see Supplementary Table 3). After exclusion, thirty-six patients were recorded four times across 6 weeks with a gap of at least 2 days (CBP = 9 ± 12 days, CM = 12 ± 19 days) between sessions. Four patients (2 CBP and 2 CM) were recorded three times.

### Experimental procedure

During four separate fMRI recordings, patients rated the intensity of their ongoing pain for 25 min using an MRI-compatible potentiometer slider. Two CBP patients and two CM patients underwent three recordings. The scale ranged from zero to 100 in steps of five with zero representing no pain and 100 representing the highest experienced pain^45^. On a screen, a moving red cursor on a dark grey bar (visual analogue scale, VAS) and a number above (numeric analogue scale, NAS) were shown during the entire functional MRI session. The screen was visible through a mirror mounted on top of the MRI head coil. Patients were asked to look only at the screen, focus on their pain with an emphasis on rising and falling pain. The intensity and the changes of perceived pain had to be indicated as quickly and accurately as possible. To minimise head movement, foams were placed around the head and patients were told to lie as still as possible.

We did not include a healthy control group. Although this group would likely show some fluctuating network activity there is no endogenous pain to relate the fluctuating network activity to. A “correlation” with pain ratings that consists of zeros is statistically not possible. Therefore, a healthy control group using this design is not applicable.

### Data acquisition

Data were recorded on a 3 T MRI scanner (Siemens Magnetom Skyra, Germany) with a 64-channel head coil. Using a multiband sequence (factor 2, T2*-weighted BOLD (blood oxygenation level dependent), images were acquired with the following parameters: number of slices = 46; repetition time/echo time = 1550/30 ms; flip angle = 71°; slice thickness = 3 mm; voxel size = 3 × 3 × 3 mm^3^; field of view = 210 mm. 1000 volumes were recorded in 1550 s (TR = 1.55 s). Field maps were acquired in each session to control for B0-effects. For each patient, T1-and T2-weighted anatomical MRI images were acquired using the following parameters for T1: repetition time/echo time = 2060/2.17 ms; flip angle = 12°; number of slices = 256; slice thickness = 0.75 mm; field of view = 240 mm, and for T2: repetition time/echo time = 3200/560 ms; flip angle = 120°; number of slices = 256; slice thickness = 0.75 mm; field of view = 240 mm. Head motion did not exceed ± 2 mm or ± 2°.

### Data processing: behavioural data

The rating data were continuously recorded with a variable sampling rate but down-sampled offline at 10 Hz. To remove the same filtering effects from the behavioural data as from the imaging data, we applied a 400 s high-pass filter (see below). For the statistical analysis, the resulting filtered time course was transferred to Matlab (Mathworks, USA, version R2018a) and down-sampled to the sampling frequency of the imaging data (1/1.55 Hz). Similar to the concept of cross-correlation, we shifted the rating vector between − 25 and 35 s in steps of 0.5 s (121 steps). These systematic shifts would account for (a) the unknown delay of the BOLD response and (b) for the unknown timing of cortical processing in reference to the rating: some ongoing cortical processes may influence later changes in pain ratings, other processes are directly related to the rating behaviour, or are influenced by the rating process and are occurring afterwards. We are aware that the variable timing of the BOLD response and the variable timing of the cortical processes are intermingled and would interpret the timing aspects with utmost caution.

To disentangle the distinct aspects of pain intensity (AMP - amplitude) from cortical processes related to the sensing of rising and falling pain, we generated a further vector by computing the ongoing rate of change (SLP - slope, encoded as 1, -1, and 0) in the pain ratings. The rate of change is represented as the slope of the regression of the least-squares line across a 3 s time window of the 10 Hz pain rating data. SLP and aSLP vectors were derived from unfiltered behavioural data. We applied the same shifting (121 steps) as for the amplitude time course. A vector of the absolute slope of pain ratings (aSLP - absolute slope, encoded as 0 and 1), represents periods of motor activity (slider movement), changes of visual input (each slider movement changes the screen), and decision-making (each slider movement prerequisites a decision to move). AMP, SLP, and aSLP vectors were convolved with a haemodynamic response function (HRF) implemented in SPM12 with the following parameters: HRF = spm_hrf(0.1,[6 16 1 1 100 0 32]). To avoid any effects of order (in case of continuously rising pain) in the data, the patients’ rating time courses were required to fluctuate at a relatively constant level. Furthermore, cortical analogues of continuously rising pain would not survive the required high-pass filtering. To ensure the behavioural task performance of the patients fulfilled these criteria, the ratings of each patient’s pain was measured with a constructed parameter PR (see Supplementary File 2).

### Data processing: imaging data

Functional MRI data were preprocessed using FSL (Version 5.0.10), which included removal of non-brain tissue (using brain extraction, BET), slice timing correction, head motion correction, B0 unwarping, spatial smoothing using a Gaussian kernel of FWHM (full width at half maximum) 6 mm, a nonlinear high-pass temporal filtering with a cutoff of 400 s, and linear and non-linear registration to the Montreal Neurological Institute (MNI) standard template. The low-pass filter setting has been determined in order to account for the signal decay due to the frequency shift of the EPI sequence (lower frequency limit of the low-pass filter) and to prevent any elimination of low-frequency changes in pain ratings and pain-related cortical data (upper frequency limit of the low-pass filter). The data were further cleaned of artefacts by performing single-subject ICA with MELODIC. ICA-based approaches are able to separate cortical sources from noise sources^46,47^. Artefact-related components were evaluated according to their spatial or temporal characteristics and were removed from the data^48^. The average number of artefact components for CM was 40 ± 6 and for CBP 49 ± 8. We deliberately did not include any correction for autocorrelation, neither for the processing of the imaging data nor for the processing of the pain rating time course as this step has the potential to destroy the natural evolution of the processes we aim to investigate (see Supplementary File 3).

### Statistical analysis: imaging data

In a next step, we ran a group ICA - separately for CBP and CM with temporally concatenated data of all recordings using MELODIC. The rationale to run separate ICAs was to find components that are tuned as much as possible to the characteristics of the patient groups. The number of components was restricted to 100. Dual regression was then used to derive the corresponding network time series and maps for all components and for each of the sessions. Using Linear Mixed Effects models (LME; MixedModels.jl package in Julia), we aimed to determine the relationship between fluctuating pain intensity and the fluctuating cortical activity separately for each component. The fluctuating network activity of a particular component is modelled through the time course of the three variables (AMP, SLP, aSLP) derived from the pain ratings.

The statistical model is expressed in Wilkinson notation; the included fixed effects (network ~ AMP + SLP + aSLP) describe the magnitudes of the population common intercept and the population common slopes for the relationship between cortical data and pain perception. The added random effects (e.g. AMP - 1 | session) model the specific intercept differences for each recording session (e.g. session-specific differences in pain levels or echo-planar image signal intensities). For a comprehensive introduction into LMEs see Harrison et al.^49^.

1. network ~ AMP + SLP + aSLP + (AMP − 1 | session) + (SLP − 1 | session) + (aSLP − 1 | session)

Each model was computed 121 times along the time shift of the rating vector (− 25 to 35 s in steps of 0.5 s, see above) and the highest absolute t-values of the fixed effect parameters AMP and SLP were extracted. Due to arbitrary units of the EPI time series, the random effects are not interpretable in this model.

We deliberately decided against a direct comparison of both pain diseases. Due to the fact that we have investigated only two pain diseases, we would not assume a specificity of these networks for any pain disease. Any outcome of the contrast between CBP and CM would give a false impression of specificity. Furthermore, the ICAs have been computed separately for both groups; the components do not overlap. Results were corrected for multiple comparisons and autocorrelation of the time series (see Supplementary File 3).

In order to confirm the correspondence of our significant networks to the known nomenclature (DMN, SN), we computed a spatial correlation of our maps (#11,#12,#17) with the homonymous network of a resting-state study^11^. Maps were thresholded at z = 1.5; data pairs were included if both voxels exhibited values beyond that threshold.

## Supplementary Information

Supplementary Information 1.

Supplementary Information 2.

Supplementary Information 3.

Supplementary Information 4.

Supplementary Information 5.

## Data Availability

The data that support the findings of this study are available from the corresponding author upon reasonable request.
