# Supplementary material for: Intrinsic network activity reflects the ongoing experience of chronic pain
Source: Sci Rep. 2021 Nov 8;11:21870. doi: 10.1038/s41598-021-01340-0 (PMC8576042; doi:10.1038/s41598-021-01340-0)
Supplement: Supplementary file 1 — Supplementary Information 1. [file 41598_2021_1340_MOESM1_ESM.pdf]

Supplementary Table 1 Characteristics of CBP patients reported in questionnaires

#	m/f	age (years)	pain duration (years)	pain location	pain medication	pain intensity	PCS	d/a/s
1	f	52	32	thoracic	none	4	14	1/4/11
2	m	64	2	lumbar	none	3	22	3/5/7
3	f	39	8	lumbar	Ibuprofen 600 mg (10-12x/month))	4	32	7/9/16
4	f	41	18	lumbar	Fluoxetine 20 mg (daily), Paracetamol 500 mg (2-3x/month), Orphenadrine 100mg (3x/month)	3	33	6/7/11
5	f	60	14	thoracic	Diclofenac 69,82 mg (1x/month)	4	14	2/5/6
6	f	39	3	lumbar	Paracetamol 500mg (2-3x/month), Ibuprofen 400mg (2x/month)	4	13	2/1/1
7	f	48	5	cervical	Ibuprofen 400mg (4x/month)	5	12	4/0/6
8	f	52	11	thoracic & lumbar	Ibuprofen 400mg (5x/month), Metamizole 500mg (2x/month)	4	7	1/2/5
9	f	31	6	lumbar	Ibuprofen 800mg (2-3x/month), Metamizole 1000mg (2-3x/month)	5	1	0/0/1
10	m	26	4	lumbar	none	5	9	0/3/3
11	f	55	16	thoracic & lumbar	Cannabis drops (15x/month)	10	11	5/2/6
12	m	65	8	lumbar	Ibuprofen 600mg (10-15x/month)	4	10	2/2/3
13	f	31	1	cervical & thoracic	Ibuprofen 400mg (18x/month)	3	25	9/6/14
14	m	32	7	thoracic and lumbar	Ibuprofen 600mg (6x/month), Tramadol 100mg (10x/month)	5	22	7/3/5
15	f	26	10	lumbar	Ibuprofen 400mg (1x/month)	4	18	6/3/8
16	f	55	16	cervical & thoracic	Metamizole 500mg (2-3x/month)	5	2	4/0/4
17	f	56	15	lumbar	none	7	36	4/4/13
18	f	42	11	thoracic & lumbar	none	5	10	1/2/6
19	f	30	3	thoracic	Ibuprofen 400mg (3x/month)	4	27	2/4/8
20	f	43	10	cervical & lumbar	Ibuprofen 400mg (5x/month)	7	22	5/4/6

m/f: male/female; PCS: pain catastrophizing scale; d/a/s: depression/anxiety/stress. The cutoff for depression and stress is 10, for anxiety 6, and for the PCS 30.

Supplementary Table 2 Characteristics of CM patients reported in questionnaires

#	m/f	age (years)	pain duration (years)	pain medication	pain intensity	PCS	d/a/s
1	f	61	50	Sumatriptan 100mg (20x/month)	7	15	0/10/9
2	f	27	7	Metamizole 500mg (2-3x/month), Sumatriptan 50mg (1x/month)	4	5	0/0/2
3	f	50	35	Sumatriptan 100mg (5-7x/month)	4	24	5/0/6
4	f	27	8	Zolmitriptan 20mg (2x/month), Ibuprofen 600mg (7x/month)	7	37	1/1/8
5	m	49	30	Ibuprofen 600mg (7-8x/month), Metamizole 500mg (3-4x/month), Paracetamol 500mg (5-6x/month)	4	3	0/6/1
6	f	52	30	Ibuprofen 400mg (6x/month), Paracetamol 1000mg (2x/month)	5	11	5/10/12
7	f	32	15	Zolmitriptan 5mg (8x/month), Naproxen 500mg (15x/month), Acetylsalicylic Acid (ASA) 250mg (4x/month), Paracetamol 200mg (4x/month), Caffeine 50mg (4x/month)	4	31	5/7/7
8	f	21	7	Sumatriptan 50mg (1x/month)	4	10	2/1/0
9	f	19	7	none	4	35	10/2/6
10	f	46	13	Ibuprofen 800mg (8-10x/month)	6	31	7/10/15
11	f	27	13	Triptan (2-3x/month), ASA 250mg (20-25x/month), Paracetamol 250mg (20-25x/month), Caffeine 50mg (20-25x/month)	4	13	6/1/10
12	m	53	15	Ibuprofen 600mg (10x/month)	6	20	7/2/4
13	f	30	6	Ibuprofen 400mg (4x/month)	4	24	1/1/5
14	f	21	7	Ibuprofen 600mg (4-8x/month), Paracetamol 500mg (4x/month), Zolmitriptan 5mg (1-2x/month)	3	15	2/0/5
15	f	23	8	Ibuprofen 600mg (2-3x/month)	7	24	2/0/3
16	f	28	7	Ibuprofen 500mg (10x/month)	7	11	0/1/1
17	f	25	5	Ibuprofen 600mg (5x/month), Zolmitriptan 5mg (1x/month)	4	21	5/5/9
18	f	33	20	Paracetamol 500mg (3x/month)	5	32	4/0/4
19	f	21	9	Ibuprofen 400mg (6-10x/month), Rizatriptan 10mg (2x/month)	5	30	1/1/2
20	f	43	10	Ibuprofen 400mg (20x/month), Paracetamol 325 mg (8-10x/month), Naproxen 100 mg (8-10x/month), Caffeine 50 mg (8-10x/month), Drotaverine hydrochloride 40 mg (8-10x/month), Pheniramine 10 mg (8-10x/month)	4	22	2/3/9

m/f: male/female; PCS: pain catastrophizing scale; d/a/s: depression/anxiety/stress. The cutoff for depression and stress is 10, for anxiety 6, and for the PCS 30.
