# Supplementary material for: Intrinsic network activity reflects the ongoing experience of chronic pain
Source: Sci Rep. 2021 Nov 8;11:21870. doi: 10.1038/s41598-021-01340-0 (PMC8576042; doi:10.1038/s41598-021-01340-0)
Supplement: Supplementary file 2 — Supplementary Information 2. [file 41598_2021_1340_MOESM2_ESM.pdf]

Supplementary document on PR parameter

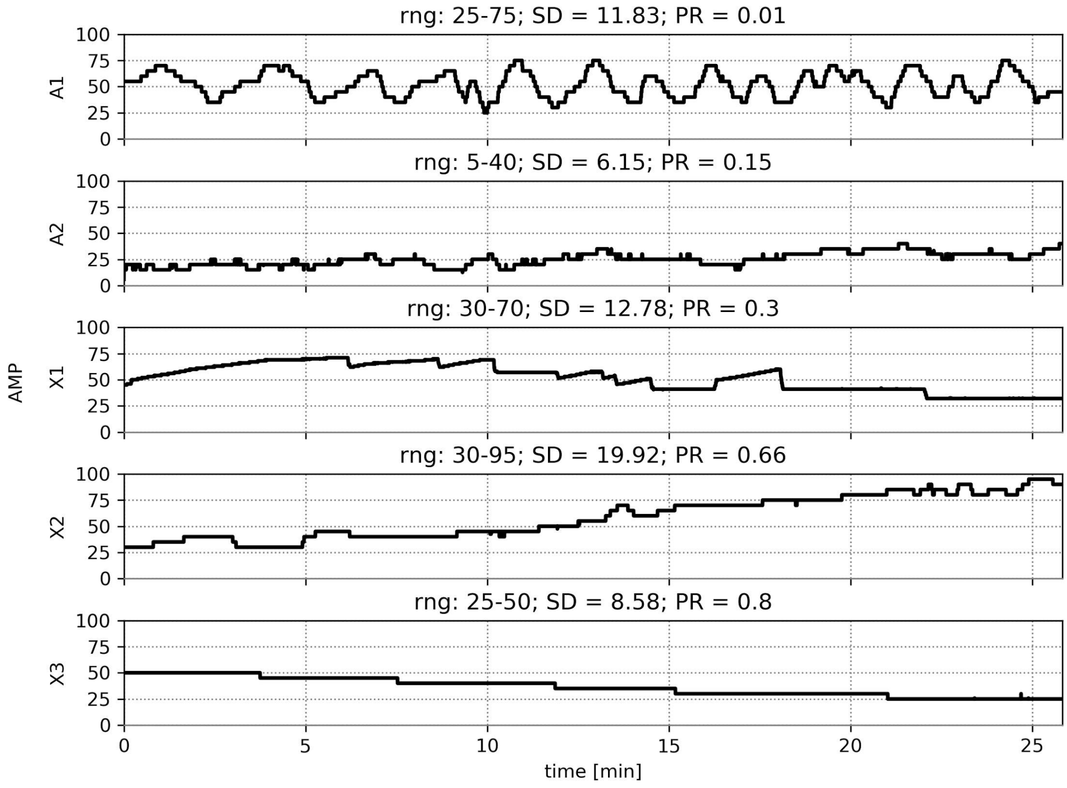

Accepted and rejected pain ratings based on the parameter PR. Rating A1 represents an excellent rating with a very low PR value of 0.01, indicating high variance and no overall drift throughout the experiment. Rating A2 represents a pain rating with a moderate PR value of 0.15, whereas X1 represents an excluded rating with a PR slightly higher than the threshold PR ≥ 0.25. X2 was excluded due to a steady increase in the pain ratings over the course of the experiment. X3 was excluded due to very low variability in ratings after high-pass filtering. Ratings A1 and A2 were accepted, whereas recordings X1, X2 and X3 were excluded from the analysis.

The ratings of each patient's pain were evaluated with the parameter PR defined as follows, with "Δpainud/Δtime" being the slope of the regression line of the unfiltered data (ud) and σfd being the sample standard deviation of the filtered data (fd):

$$(1)\ PR = \left| \frac{\Delta pain_{ud} / \Delta time}{\sigma_{fd}} \right|$$

We constructed the PR parameter through logical reasoning and theoretical considerations in a way that its minimisation is desirable. The numerator describes how much the prerequisite is violated by fitting a least squares line across the rating time course. This violation can be compensated if the variance of pain ratings is at least four times higher than the slope of the regression of the least squares line across the entire time course of the pain ratings. The standard deviation of the filtered data expressed in the denominator gives a measure of the desired fluctuation of the pain ratings but is stripped from a potential trend throughout the experiment. A minimisation of the quotient is given either

by minimising the numerator corresponding to a small increase of the overall pain ratings over the whole experiment (small slope) or by maximising the denominator corresponding to a large variability in pain ratings across the rating task. Minor overall rising in pain intensity over the whole time of the experiment could be compensated by a greater variance of ratings; small fluctuation of pain intensity would only be accepted in cases of minor pain rating trends across the entire experiment. Recordings showing PR values of ≥0.25 were rejected from the analysis or repeated if possible. We excluded five participants and repeated three sessions. The threshold of the PR value was chosen based on theoretical considerations (effect of order, data filtering) as well as on a careful inspection of the data.

Please note that subjects with a *high PR* value are *not suitable* for a continuous pain rating design for two reasons. Firstly, steadily changing ratings will cause an effect of order. Secondly, the main changes of brain activity (see X2 and X3 in this figure) will be removed from the data due to the necessary high-pass filtering. There is no literature we could have relied on in this matter.
