# Supplementary material for: Intrinsic network activity reflects the ongoing experience of chronic pain
Source: Sci Rep. 2021 Nov 8;11:21870. doi: 10.1038/s41598-021-01340-0 (PMC8576042; doi:10.1038/s41598-021-01340-0)
Supplement: Supplementary file 3 — Supplementary Information 3. [file 41598_2021_1340_MOESM3_ESM.pdf]

Correcting statistical testing - surrogate data

All statistical tests had to be corrected for multiple testing (components, time shifts) and autocorrelation in the behavioural data. We created 1000 surrogate time courses using the IAAFT algorithm (Iterative Amplitude Adjusted Fourier Transform) from the original rating data, which were uncorrelated to the original rating data but had the same autocorrelation structure, amplitudes, and variance as the original data [1]. In other words, we preserved the main features of the data but randomised the timing. The main features of the behavioural data were then unrelated to the time course of the imaging data. The approach has been previously utilised for the analysis of time series data [2–5]. For a more detailed explanation see Spisak (2014).

Using surrogate data, the entire LME analysis, including the temporal shifts, was repeated 1000 times, resulting in 1000*121*100 statistical tests (surrogates*shifts*ICA components) for AMP and SLP. The highest absolute t-values of each of the 1000 repetitions across all components and shifts were extracted. This procedure resulted in a right-skewed distribution of 1000 values for each condition. Based on the distributions of 1000 values (for AMP, SLP), the statistical thresholds were determined using the "palm_datapval.m" function publicly available in PALM [6,7]. The components were considered as "significant" if they exceeded the threshold provided by PALM ($p<0.05$).

These steps have an advantage compared to a potential control group of healthy subjects and a resting-state recording with random pain ratings or a paradigm with applied pain. The surrogate vectors are perfectly matched to the original vector in terms of amplitude, variance, and frequency but unrelated to the amplitude of the components' time courses.

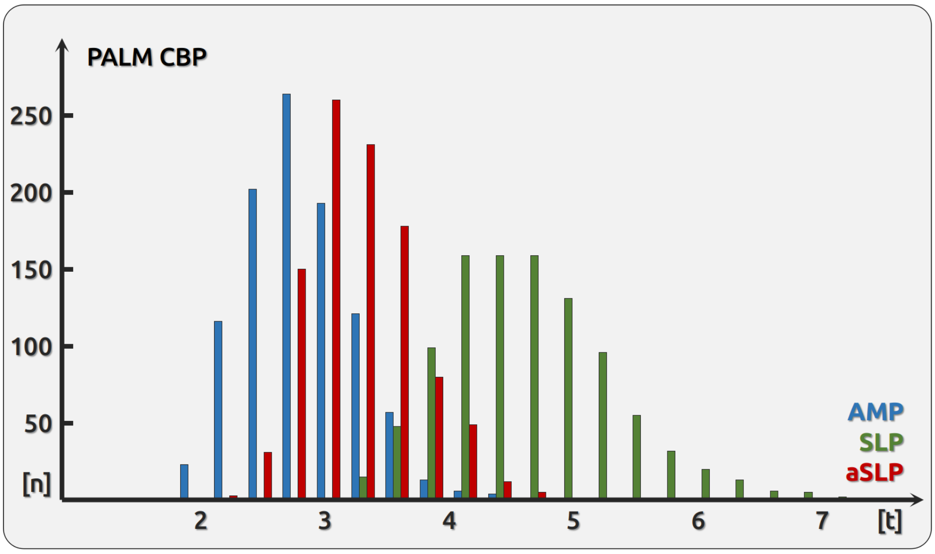

Figure 1 | Distribution of randomised statistics on surrogate data. The distributions show the absolute maximal values for each of the 1000 randomisations for CBP.

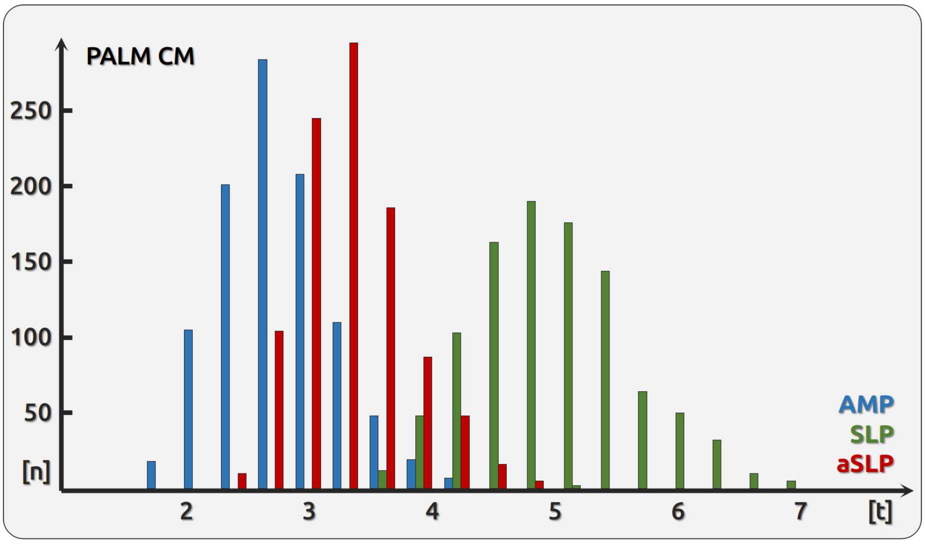

Figure 2 | Distribution of randomised statistics on surrogate data. The distributions show the absolute maximal values for each of the 1000 randomisations for CM.

References

1. Schreiber, T. & Schmitz, A. Improved Surrogate Data for Nonlinearity Tests. *Phys. Rev. Lett.* **77**, 635–638 (1996).
2. Hlinka, J., Palus, M., Vejmelka, M., Mantini, D. & Corbetta, M. Functional connectivity in resting-state fMRI: is linear correlation sufficient? *Neuroimage* **54**, 2218–2225 (2011).
3. Hindriks, R. *et al.* Can sliding-window correlations reveal dynamic functional connectivity in resting-state fMRI? *Neuroimage* **127**, 242–256 (2016).
4. Spisák, T. Individual functional statistical parametric networks related to interictal epileptic EEG discharges: a dynamic sliding-window study. (2014) doi:10.1594/ECR2014/C-2088.
5. Handwerker, D. A., Roopchansingh, V., Gonzalez-Castillo, J. & Bandettini, P. A. Periodic changes in fMRI connectivity. *Neuroimage* **63**, 1712–1719 (2012).
6. Winkler, A. M., Ridgway, G. R., Webster, M. A., Smith, S. M. & Nichols, T. E. Permutation inference for the general linear model. *Neuroimage* **92**, 381–397 (2014).
7. Winkler, A. M. *et al.* Non-parametric combination and related permutation tests for neuroimaging. *Hum. Brain Mapp.* **37**, 1486–1511 (2016).

Examples of corresponding component vs. rating time courses

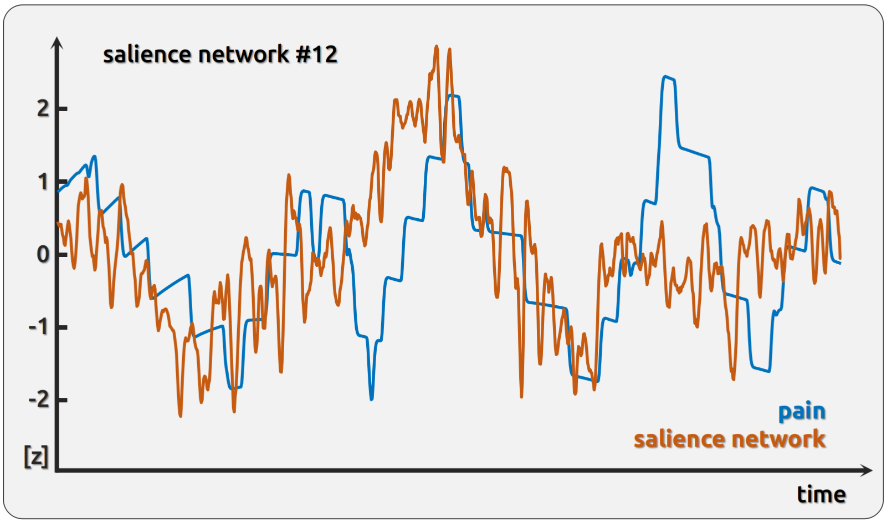

Figure 3 | Example of joint component vs. rating time courses for a single subject. For display reasons network and rating time courses were z-transformed.

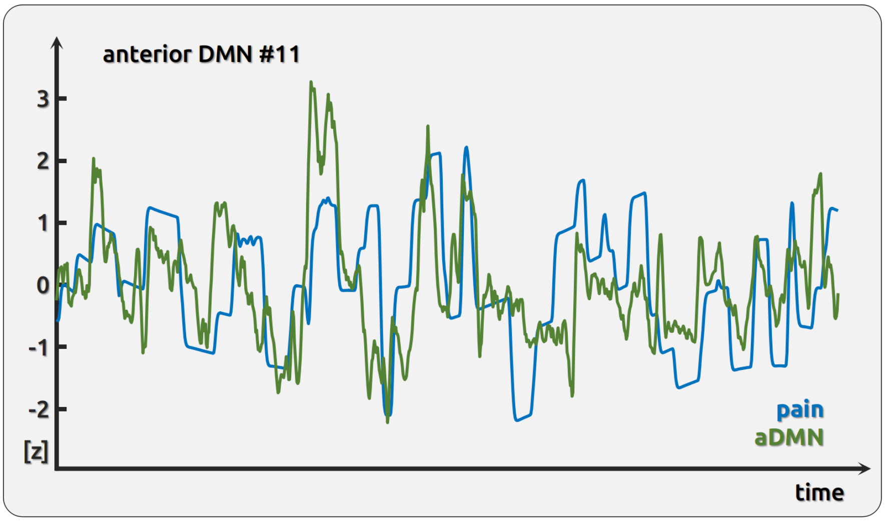

Figure 4 | Example of joint component vs. rating time courses for a single subject. For display reasons network and rating time courses were z-transformed. Due to the negative relationship between network amplitude and rating, the time course of the component has been flipped.

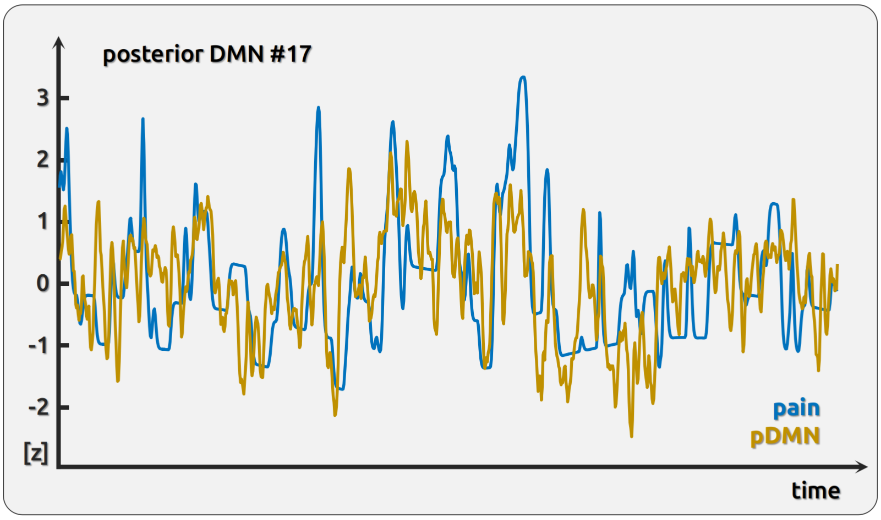

Figure 5 | Example of joint component vs. rating time courses for a single subject. For display reasons network and rating time courses were z-transformed. Due to the negative relationship between network amplitude and rating, the time course of the component has been flipped.
