# Supplementary material for: Intrinsic network activity reflects the ongoing experience of chronic pain
Source: Sci Rep. 2021 Nov 8;11:21870. doi: 10.1038/s41598-021-01340-0 (PMC8576042; doi:10.1038/s41598-021-01340-0)
Supplement: Supplementary file 4 — Supplementary Information 4. [file 41598_2021_1340_MOESM4_ESM.pdf]

#	**Exclusion criterion**
2	Development of additional pain, unrelated to the investigated pain disease (CBP)
2	Unable to comply with study request, discontinued after 1 or 2 recordings (CBP)
5	continuously increasing or decreasing pain ratings throughout the pain rating experiment (3x CM, 2x CBP)

Supplementary Table 3. A total of 9 patients were excluded.
