# Supplementary material for: Intrinsic network activity reflects the ongoing experience of chronic pain
Source: Sci Rep. 2021 Nov 8;11:21870. doi: 10.1038/s41598-021-01340-0 (PMC8576042; doi:10.1038/s41598-021-01340-0)
Supplement: Supplementary file 5 — Supplementary Information 5. [file 41598_2021_1340_MOESM5_ESM.pdf]

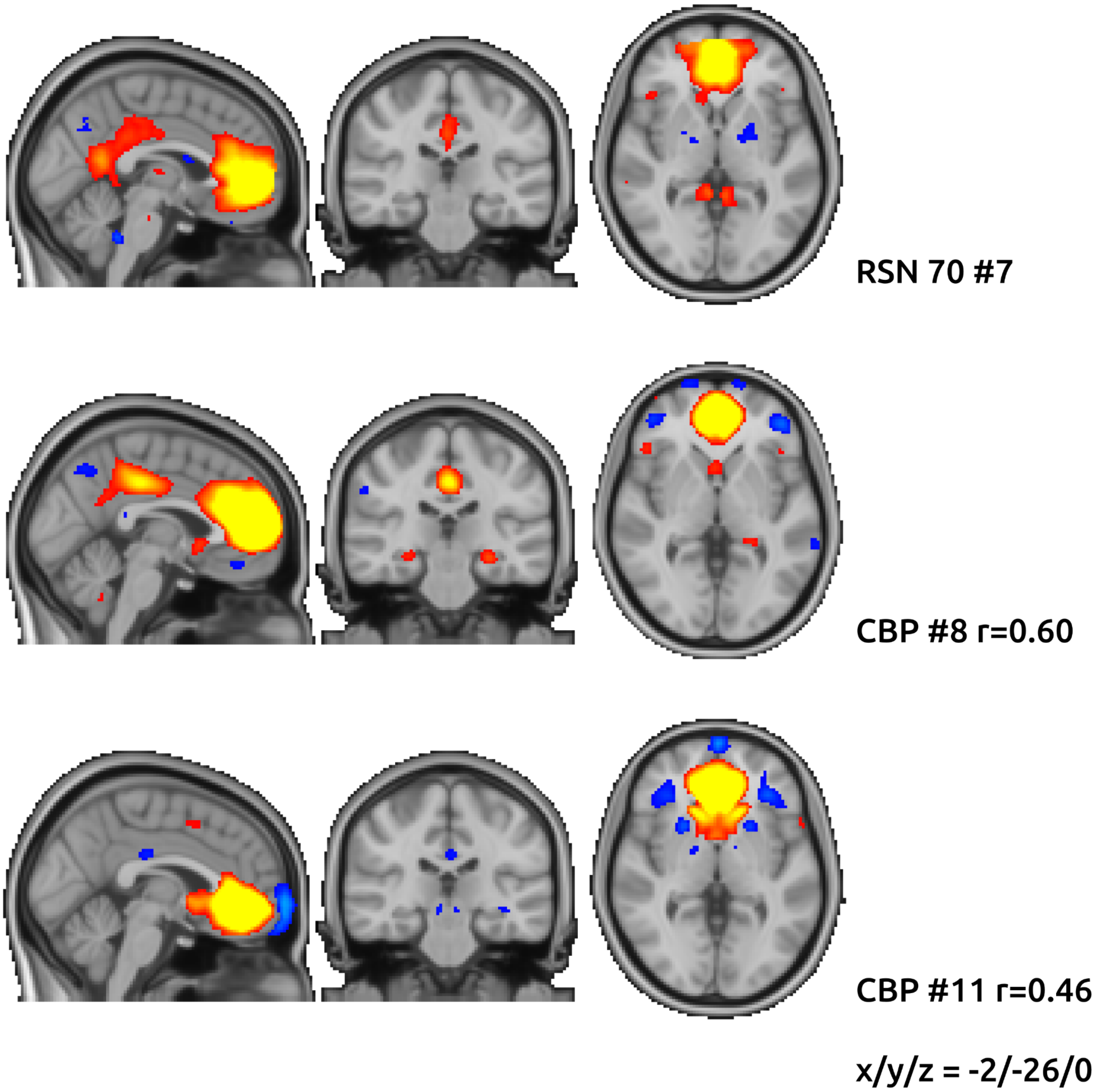

Supplementary Figure 1. | Spatial Correlation of the aDMN (Smith et al., 2009) with CBP topographies. The anterior DMN component is correlated with two components from CBP. Both are negatively correlated with pain intensity but only component 11 is significant (Figure 1).

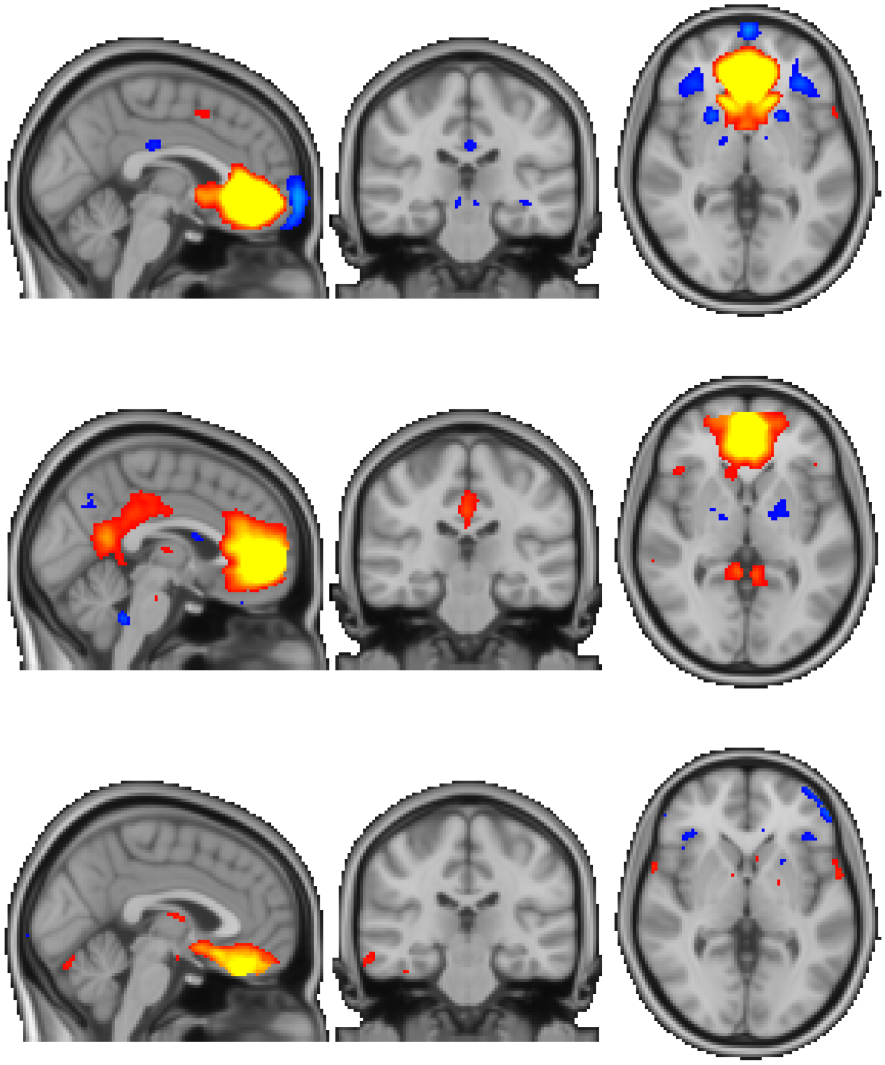

CBP #11

RSN 70 #7 r=0.46

RSN 70 #60 r=0.27

x/y/z = -2/-26/0

Supplementary Figure 2 | Correlation between the significant CBP aDMN and all RSN components (Smith et al., 2009). The only convincing spatial correlation is the one from Supplementary Figure 1(component #7). The next best correlation (component #60) has clearly a distinct topography and a much lower correlation coefficient. The figure confirms that component #11 can be considered as aDMN.

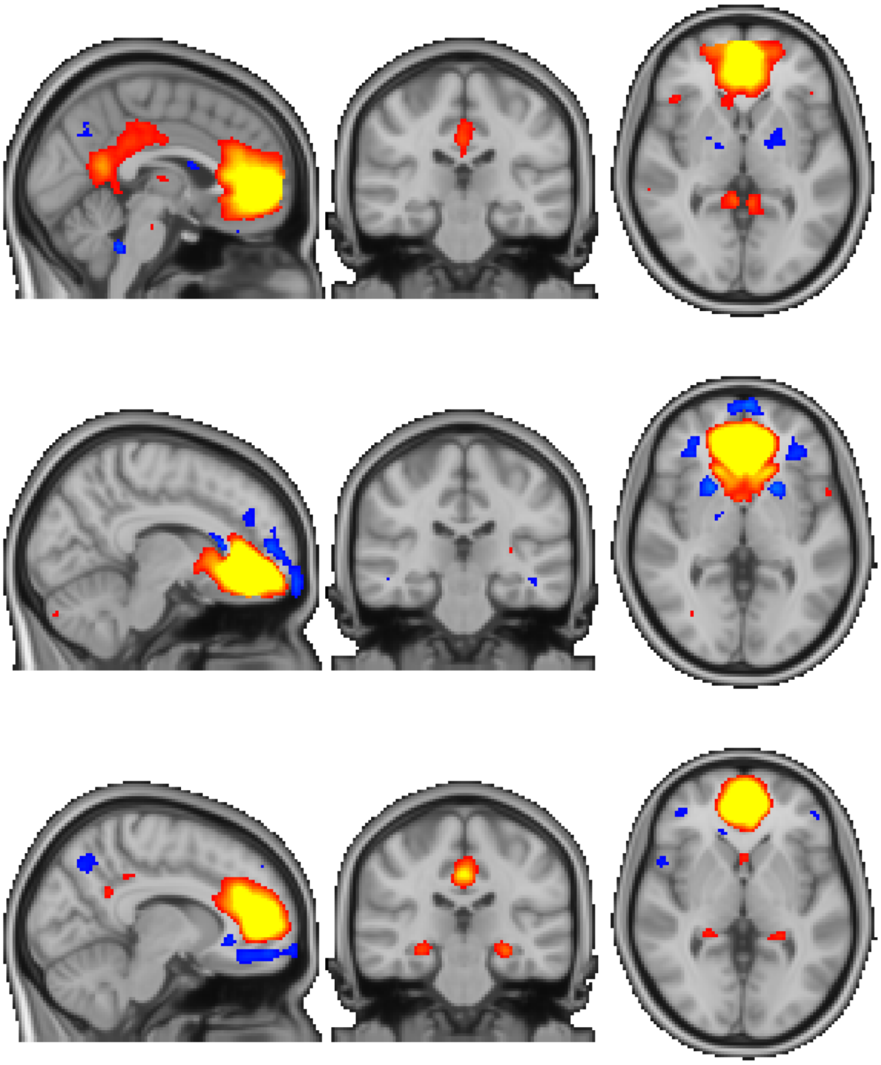

RSN 70 #7

CM #11 r=0.50

CM #15 r=0.61

x/y/z = -2/-26/0

Supplementary Figure 3 | Correlation between the aDMN (Smith et al., 2009) and all CM components. Similar to CBP we found two aDMN components. Both were negatively correlated with pain but not significant. The figure confirms that similar aDMNs exist for CM as for CBP.

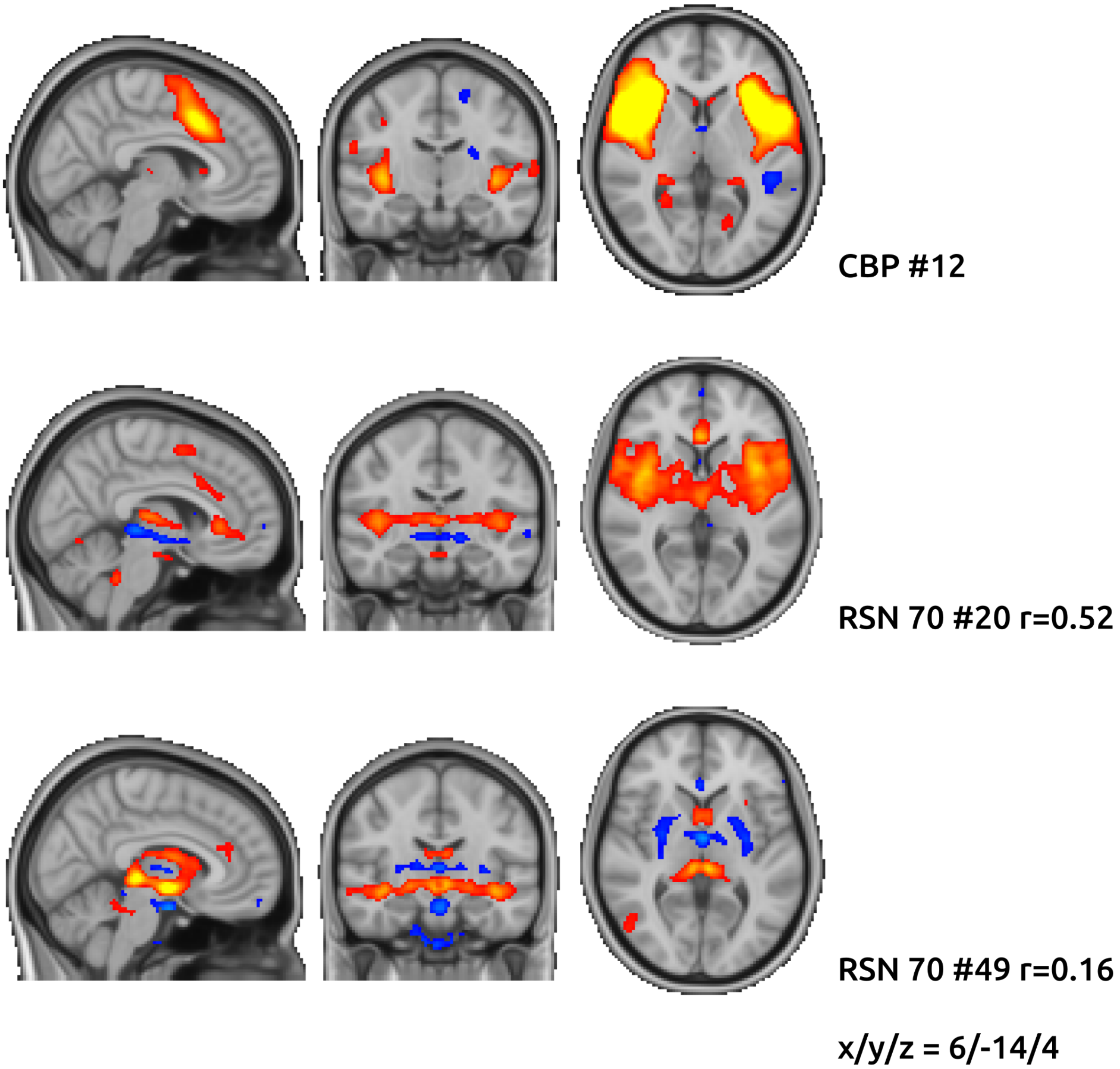

Supplementary Figure 4 | Correlation between the SN for CBP with all RSN components (Smith et al., 2009). We found only one spatially correlated component (RSN #20). The next best correlated component is probably related to CSF signals (RSN #49). The figure confirms that component #12 represents the SN.

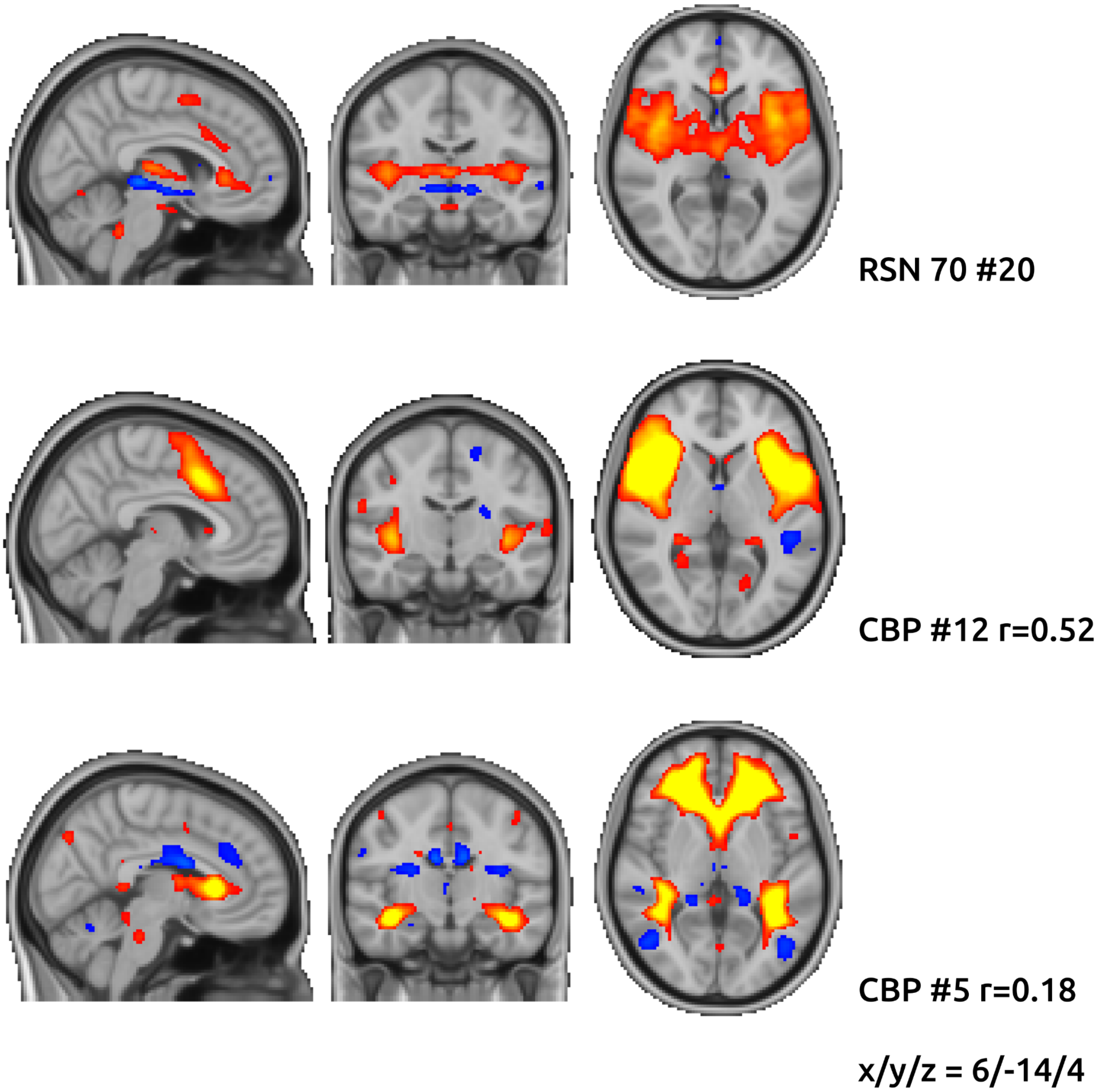

Supplementary Figure 5 | Correlation with the SN component (Smith et al., 2009) with all CBP components. We found one spatial correlation with component 12 (Figure 4). The next best correlated component is probably related to WM signal fluctuations.

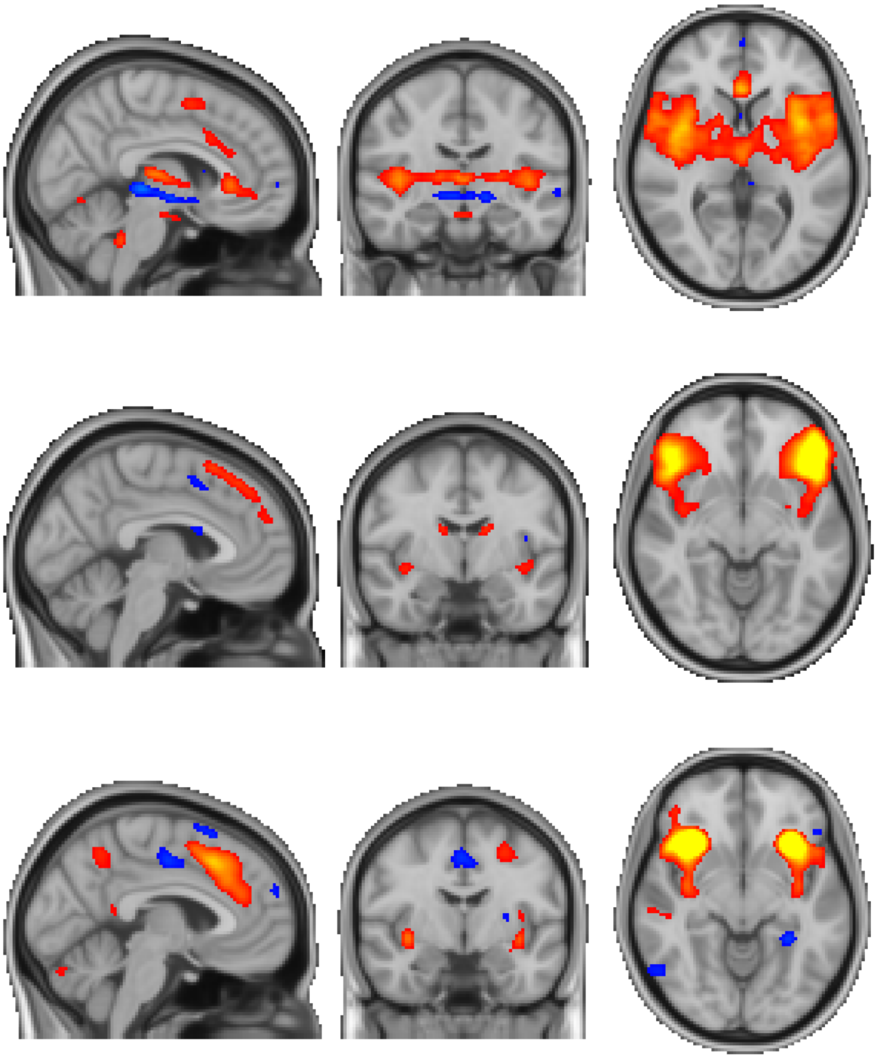

RSN 70 #20

CM #42 r=0.30

CM #34 r=0.29

x/y/z = 6/-14/4

Supplementary Figure 6 | Correlation between the SN component (Smith et al, 2009) with all CM components. The main region for #42 is located in the posterior part of the orbitofrontal cortex. The second component #34 could be interpreted as a salience network but has a slightly shifted topography. Both components for CM are unrelated to pain intensity.

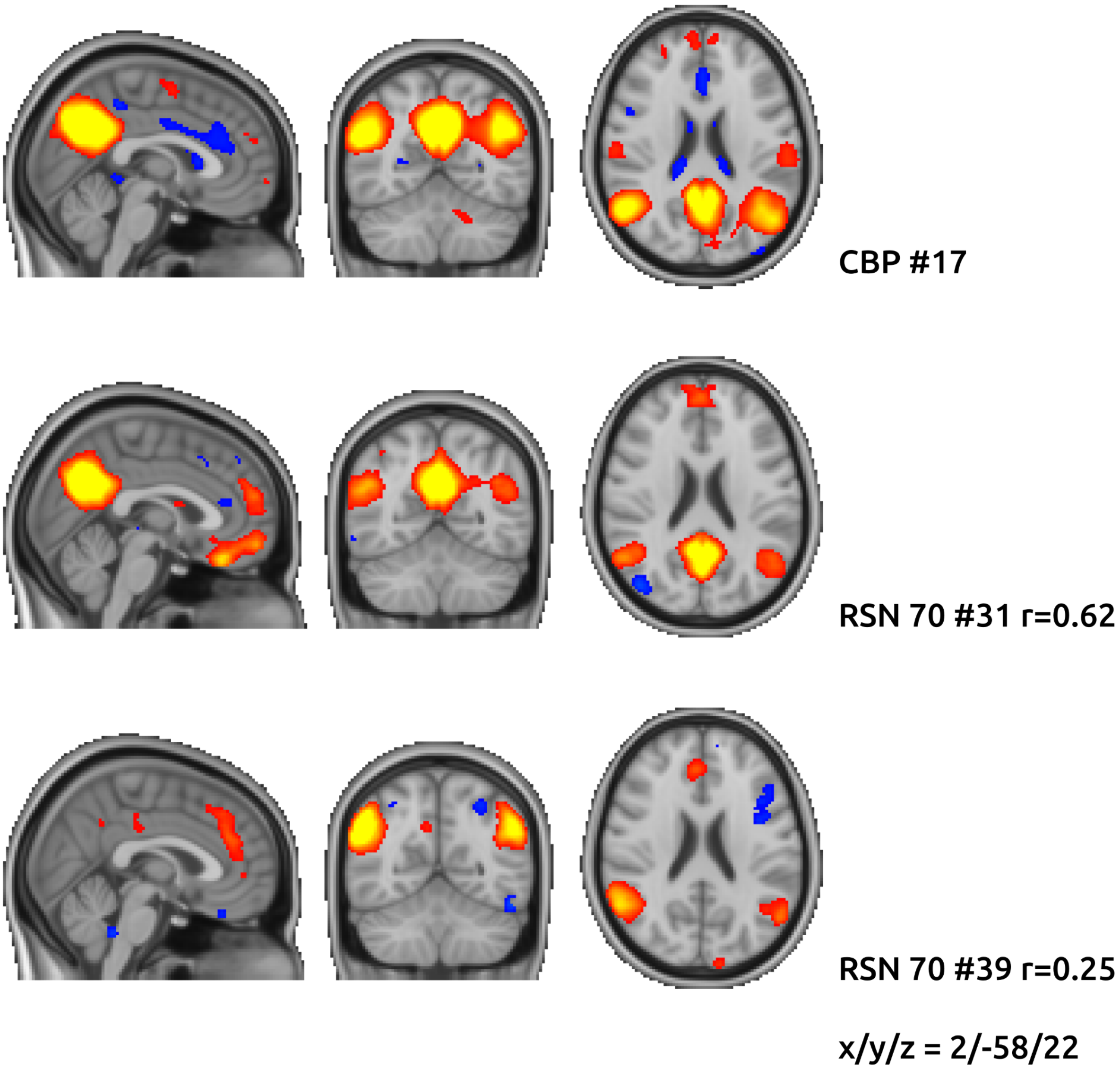

Supplementary Figure 7 | Correlation between the significant CBP pDMN and all RSN components (Smith et al., 2009). The only convincing spatial correlation is the #31. The second best correlation (component #39) has clearly a distinct topography. The spatial correlation underlines the labeling of component #17 as pDMN.

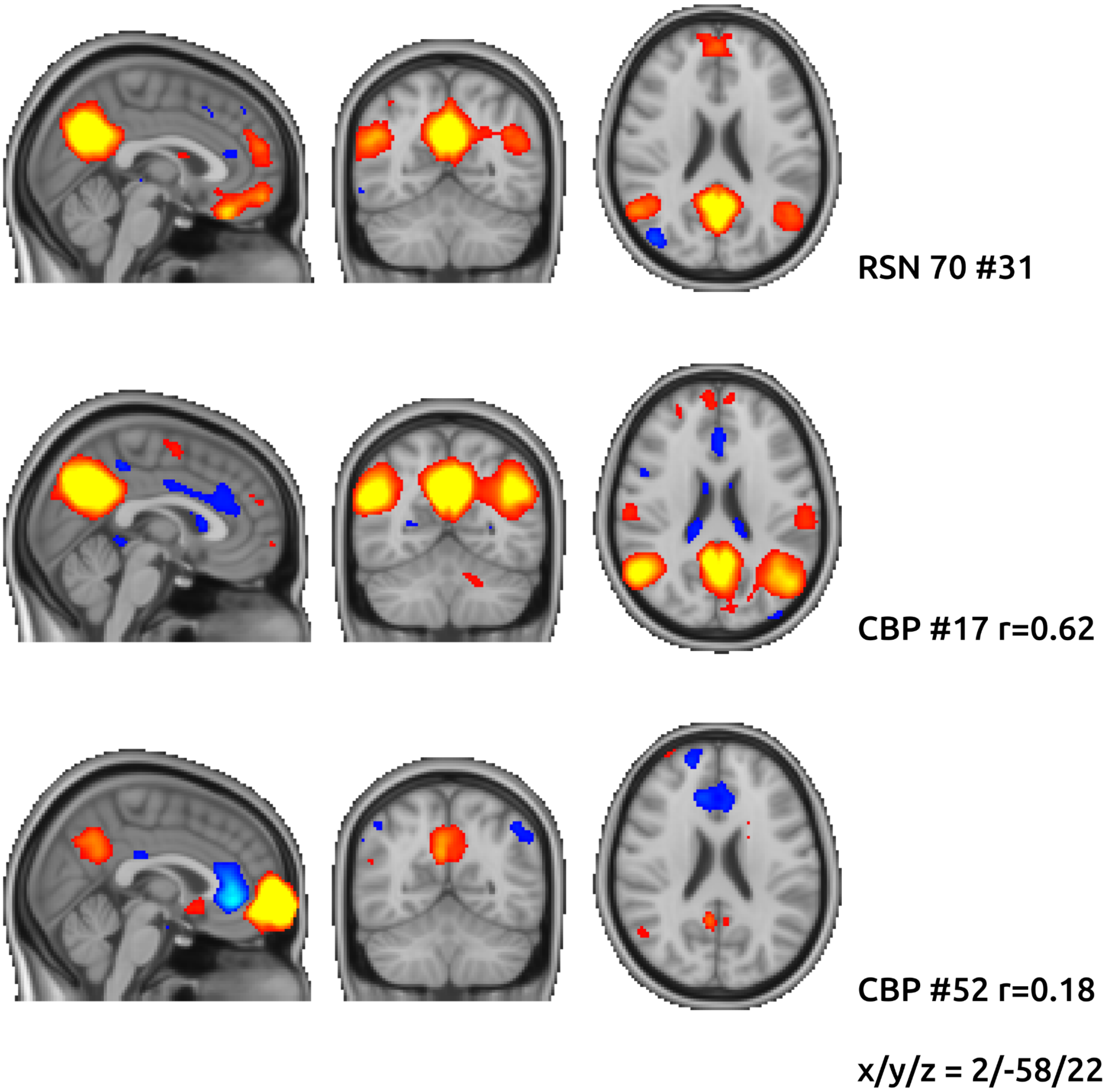

Supplementary Figure 8. | Correlation of the pDMN (Smith et al., 2009) with all CBP topographies. The posterior DMN component from the Smith study is only correlated with #17. The other component #52 has a different topography.

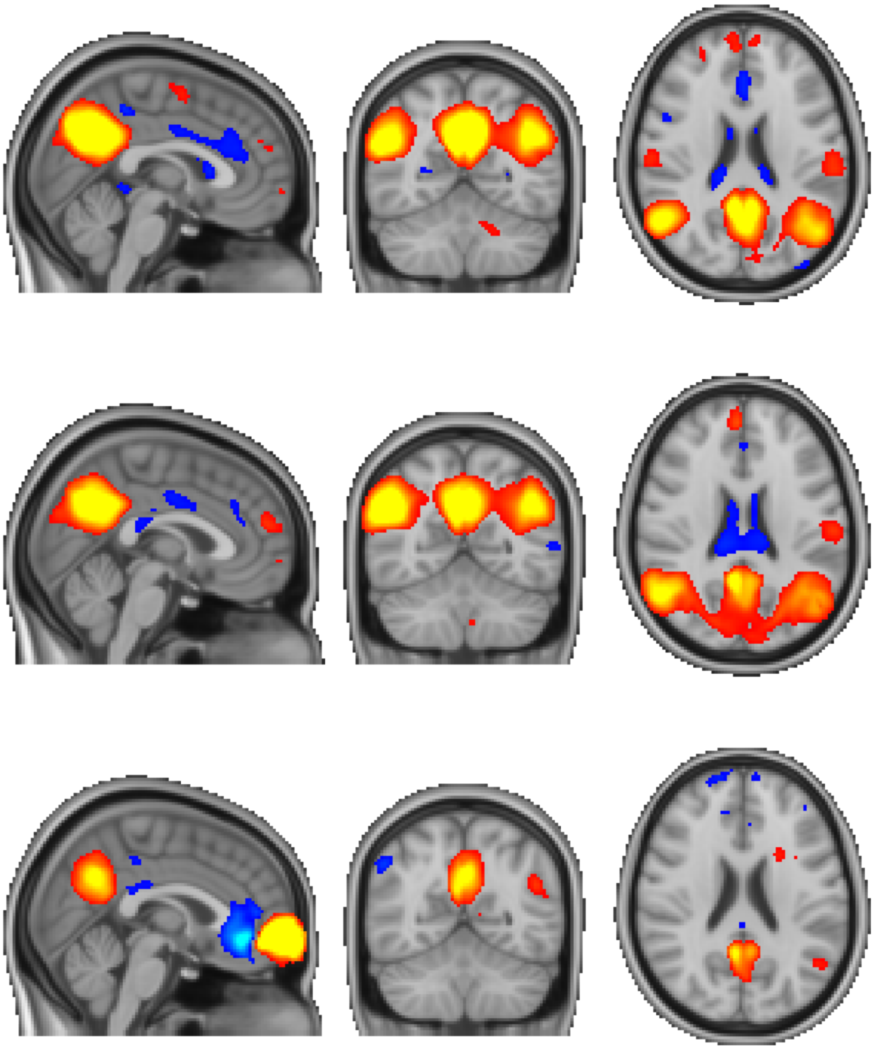

Supplementary Figure 9. | Correlation of the pDMN (Smith et al, 2009) with CM topographies. The posterior DMN component from the Smith study is only correlated with #23. The other component #58 has a different topography.

Smith et al., (2009): Correspondence of the brain's functional architecture during activation and rest. PNAS.

RSN files available at https://www.fmrib.ox.ac.uk/datasets/brainmap+rsns/rsn70.nii.gz

Matlab code

```
cmp_ICA = 17;
cmp_RSN = 23;

ICA= niftiread('/path_to_file/CBP/melodic_IC.nii.gz');
rsn= niftiread('/path_to_file/rsn70.nii.gz');

% pDMN
rsn_pDMN = squeeze(rsn(:,:,:,cmp_ICA));
rsn_pDMN(rsn_pDMN==0)=nan;

ICA_pDMN = squeeze(ICA(:,:,:,cmp_RSN));
ICA_pDMN(ICA_pDMN==0)=nan;

correlation = corr(rsn_pDMN(:),ICA_pDMN(:),'rows','complete')
```
